# EFFECTS OF SMART AMBIENT BRIGHT LIGHT (SABL) ON SLEEP QUALITY IN NURSING HOME RESIDENTS WITH DEMENTIA

**DOI:** 10.1093/geroni/igae098.2356

**Published:** 2024-12-31

**Authors:** Chia Jou Lin, Yo-Jen Liao, Diane Berish, Ying-Ling Jao

**Affiliations:** Taipei Medical University, Taipei, Taipei, Taiwan (Republic of China); The Pennsylvania State University, University Park, Pennsylvania, United States; The Pennsylvania State University, University Park, Pennsylvania, United States; Pennsylvania State University, University Park, Pennsylvania, United States

## Abstract

Lighting interventions (LIs) have shown positive impact on circadian rhythms and sleep quality in people with dementia. Yet, few studies incorporated daylight with LI. The Smart Ambient Bright Light (SABL) is a newly developed LI that accommodates natural daylight and provides automatic bright light during the day and dim light during the night. The study examined the effect of SABL on sleep in nursing home (NH) residents with dementia. This was a 13-week cluster randomized crossover-controlled trial conducted in two nursing homes in Pennsylvania. The SABL was installed in residents’ bedrooms and common areas for 4 weeks with 2 weeks of washout between control and intervention. The Pittsburgh Sleep Quality Index (PSQI) was used to measure sleep quality. Descriptive analysis and paired t-test was performed to compare difference in sleep quality changes between control and intervention periods. A total of 18 residents enrolled in this study. Participants were 87.3 years old on average and 12 of them (67%) were female. The included residents experienced poor sleep quality at baseline (mean PSQI=6.67, SD± 3.07). A paired t-test showed that comparing to the baseline, the PSQI level decreased in both intervention group (5.28 ± 3.58) and control group (4.28 ± 2.44), without statistical significance (p = 0.29). The results suggest that the SABL did not show significant effect on sleep quality among NH residents with dementia. This is a pilot study with a small sample size. More research is needed to replicate the study on a larger scale.

